# Increased parietal circuit-breaker activity in delta frequency band and abnormal delta/theta band connectivity in salience network in hyperacusis subjects

**DOI:** 10.1371/journal.pone.0191858

**Published:** 2018-01-25

**Authors:** Jae Joon Han, Ji Hye Jang, Dirk De Ridder, Sven Vanneste, Ja-Won Koo, Jae-Jin Song

**Affiliations:** 1 Department of Otorhinolaryngology-Head and Neck Surgery, Seoul National University Bundang Hospital, Seongnam, Korea; 2 Department of Surgical Sciences, Section of Neurosurgery, Dunedin School of Medicine, University of Otago, Dunedin, New Zealand; 3 Lab for Clinical and Integrative Neuroscience, School for Behavioral and Brain Sciences, University of Texas at Dallas, Richardson, Texas, United States of America; University of Zurich, SWITZERLAND

## Abstract

Recent studies have suggested that hyperacusis, an abnormal hypersensitivity to ordinary environmental sounds, may be characterized by certain resting-state cortical oscillatory patterns, even with no sound stimulus. However, previous studies are limited in that most studied subjects with other comorbidities that may have affected cortical activity. In this regard, to assess ongoing cortical oscillatory activity in idiopathic hyperacusis patients with no comorbidities, we compared differences in resting-state cortical oscillatory patterns between five idiopathic hyperacusis subjects and five normal controls. The hyperacusis group demonstrated significantly higher electrical activity in the right auditory-related cortex for the gamma frequency band and left superior parietal lobule (SPL) for the delta frequency band versus the control group. The hyperacusis group also showed significantly decreased functional connectivity between the left auditory cortex (AC) and left orbitofrontal cortex (OFC), between the left AC and left subgenual anterior cingulate cortex (sgACC) for the gamma band, and between the right insula and bilateral dorsal anterior cingulate cortex (dACC) and between the left AC and left sgACC for the theta band versus the control group. The higher electrical activity in the SPL may indicate a readiness of “circuit-breaker” activity to shift attention to forthcoming sound stimuli. Also, because of the disrupted salience network, consisting of the dACC and insula, abnormally increased salience to all sound stimuli may emerge, as a consequence of decreased top-down control of the AC by the dACC and dysfunctional emotional weight attached to auditory stimuli by the OFC. Taken together, abnormally enhanced attention and salience to forthcoming sound stimuli may render hyperacusis subjects hyperresponsive to non-noxious auditory stimuli.

## Introduction

Hyperacusis is an auditory symptom characterized by abnormal hypersensitivity to ordinary environmental sounds [1]. Patients who have hyperacusis show exaggerated or inappropriate responses to sounds that are not uncomfortable to a typical person [2]. Hyperacusis can be differentiated from phonophobia in that the latter is an intolerance and fear of specific sounds while the former is a generalized intolerance to environmental sound. The prevalence of hyperacusis has been reported to be 8–15.2% [3, 4]. In many cases, hyperacusis is associated with other clinical conditions involving the peripheral auditory system or central nervous system [5]. Peripheral neurological diseases involving facial nerve dysfunction, such as Ramsay-Hunt syndrome and Bell’s palsy, can induce deterioration of the stapedial reflex and intolerance to sound stimuli. About 86% of patients with a primary complaint of hyperacusis had combined tinnitus [6], and about 40% of tinnitus patients had simultaneous hyperacusis [7]. Additionally, migraine, depression, head injury, and William’s syndrome have been associated with hyperacusis [5]. In this regard, idiopathic hyperacusis can be defined when a patient has normal hearing thresholds without any other otologic symptom, such as tinnitus [8], and no clinical condition in the peripheral or central nervous system.

Various explanations for the pathophysiology of hyperacusis have been advanced. In some medical conditions, such as Williams syndrome, migraine, depression, and post-traumatic stress disorder, 5-HT, which modulates auditory gain and determines the significance of sound, has been suggested to contribute to the generation of hyperacusis [4]. Another hypothesis for hyperacusis is dysfunction in the outer hair cells that modulate the cochlea’s response to sound [9].

More recently, some studies have tried to explain hyperacusis as a “plasticity disease,” caused by maladaptive plasticity that leads to hyperactivation of the nervous system [10, 11]. Animal models have demonstrated hyperactivity of the auditory cortex (AC) and an exaggerated acoustic startle response after salicylate injection or noise exposure [12–14].

Moreover, human functional imaging studies have shown plastic changes in the central nervous system in hyperacusis [15]. Functional magnetic resonance imaging (fMRI) studies exploring sound-evoked activation [16] or resting-state activity [14] demonstrated increased activity in the inferior colliculus, medial geniculate body, and auditory cortical region, which was associated with decreases in loudness discomfort levels. This hyperreactivity was highly connected to the amygdala, reticular formation, and hippocampus, brain regions that are associated with fear and anxiety responses [14]. In another study using resting-state quantitative electroencephalography (rs-qEEG), increased cortical activity in the dorsal anterior cingulate cortex (dACC) and orbitofrontal cortex (OFC) was found in tinnitus patients with combined hyperacusis during resting-state recordings [17].

Although many previous studies have suggested possible explanations for the pathophysiology of hyperacusis, these studies are limited in that they did not look exclusively at hyperacusis but also included comorbidities, such as William’s syndrome and tinnitus. For this reason, determining the pathophysiology of idiopathic isolated hyperacusis *per se* has been problematic. Thus, in this study, we sought to further understand the ongoing pathognomonic cortical oscillatory activity in patients with idiopathic hyperacusis with no comorbid audiological or neurological symptoms or signs. We hypothesized that resting-state cortical activities in hyperacusis patients would show patterns differing from those of normal controls. [17]In particular, we hypothesized that components of the salience network [18, 19], a brain network that is activated only when behaviorally relevant information is processed [18, 20] might be involved in subjects with hyperacusis as components of the salience network has been shown to be activated in aforementioned previous study of ours [17].

To demonstrate this, we investigated the resting-state cortical activity in five idiopathic hyperacusis patients who had no other audiological symptoms (e.g., hearing loss, tinnitus) or other neurologic disorder. Using rs-qEEG, we show resting-state cortical oscillatory patterns of hyperacusis subjects that differ from those of age- and sex-matched normal controls. Most studies have focused on sound stimuli-driven neural activity changes, in animal models or human subjects with hyperacusis. In contrast, in the current study, we sought to reveal resting-state (pre-sound stimulus state) features of cortical oscillatory patterns in hyperacusis subjects that may explain their hyperresponsiveness to forthcoming sound stimuli.

## Materials and methods

### Participants

In total, five patients with idiopathic hyperacusis were enrolled. The subjects had no other otological symptoms, such as tinnitus or hearing loss. Patients who had neurological or psychiatric disorders, chronic headaches, drug/alcohol abuse, current psychotropic or central nervous system-activating medications, or a history of head injury with loss of consciousness or seizures were excluded. The median pure tone threshold was 5.8 dB (range: 0–22.5 dB) on the right side and 5.0 dB (range: 0–21.7 dB) on the left side (Table 1).

**Table 1:** Patients and controls’ demographic characteristics.

Subject number	Age	Sex	Right hearing threshold (dB HL)	Left hearing threshold (dB HL)	Nature of hyperacusis sound
Patient 1	60	Female	22.5	21.7	loud and echoing sound
Patient 2	30	Female	5.8	5.0	loud and echoing sound
Patient 3	19	Female	0.0	0.0	sound with noise
Patient 4	20	Male	9.2	7.5	sound with pain
Patient 5	30	Male	4.2	2.5	sound with pain
Control 1	60	Female	20.0	20.0	NA
Control 2	30	Female	10.0	10.0	
Control 3	19	Female	5.0	5.0	
Control 4	20	Male	5.0	5.0	
Control 5	30	Male	10.0	10.0	

NA, not applicable; HL, hearing level.

Using one-to-one matching, five subjects of the same sex and age, with no audiological symptoms such as hyperacusis, tinnitus, or hearing loss, were selected as a control group from a normative database consisting of 231 participants who underwent rs-qEEG analyses. This study was approved by the Institutional Review Board of Seoul National University Bundang Hospital for research involving human subjects (No. B-1606/350-301). All subjects gave written informed consent in accordance with the Declaration of Helsinki.

### EEG recording and preprocessing

EEGs were recorded for ~5 min under eyes-closed resting-state without any sound stimuli at 19 scalp sites using a tin electrode cap (Electro-Cap, Eaton, OH, USA) connected to a Mitsar amplifier (Mitsar EEG-201; Mitsar, St. Petersburg, Russia), and the data were saved using WinEEG software (ver. 2.84.44; Mitsar) (available at: http://www.mitsar-medical.com). EEG recordings were conducted in a fully lit room that was shielded from sound and stray electric fields while the patients sat upright on a comfortable chair. While recording the EEG stream, impedances at all electrodes were maintained below 5 kΩ. Data were recorded at a sampling rate of 1,024 Hz and then filtered using a high-pass filter of 0.15 Hz and a low-pass filter of 200 Hz. After initial data acquisition, off-line data processing was performed initially with resampling to 128 Hz and then band-pass filtering using a fast Fourier transform filter and application of a Hanning window at 2–44 Hz; then, the data were imported into the Eureka! software [21]. All episodic artifacts, including eye blinks, eye movements, teeth clenching, or body movements were inspected manually and removed from the EEG stream with the Eureka! software. Further artifact removal was performed by independent component analysis using the ICoN software (http://sites.google.com/site/marcocongedo/software/nica) [22, 23].

Alcohol and caffeinated beverages were prohibited for 24 h prior to EEG recording to avoid alcohol-induced changes in the EEG stream [24] or caffeine-induced alpha and beta power decreases [25]. The vigilance of participants was monitored with EEG parameters, such as slowing of the alpha rhythm and appearance of spindles, to prevent possible enhancement of theta power [26]; the included participants showed no such drowsiness-related EEG changes. The whole process, from the initial EEG recording to the manual and software-assisted artifact removal steps, have been described in previous reports [17, 27–32].

### Source localization analysis

The localization of the intracerebral sources from the recorded EEG streams was performed with low-resolution brain electromagnetic tomography (LORETA)-KEY software (available at http://www.uzh.ch/keyinst/NewLORETA/Software/Software.htm, version 20151222), a functional imaging toolbox dedicated to the functional localization of standardized current densities, based on electrophysiological and neuroanatomical constraints [33]. Standard electrode positions were registered and no regularization was performed for the transformation matrix. Group comparison between the hyperacusis group and the control group were performed using log-transformed sLORETA data in the frequency domain (i.e., FFT current density analysis) for whole-brain using t-statistics. Besides the log-transformation, no normalization or any other transformation of variables was performed. The source localization was performed based on each of the following eight frequency bands: delta (2–3.5 Hz), theta (4–7.5 Hz), alpha1 (8–10 Hz), alpha2 (10–12 Hz), beta1 (13–18 Hz), beta2 (18.5–21 Hz), beta3 (21.5–30 Hz), and gamma (30.5–44 Hz) [17, 27–32, 34, 35]. We have included gamma frequency band as we have successfully demonstrated robust gamma power in our previous reports [28, 36]. The LORETA-KEY algorithm tries to solve the inverse problem (source reconstruction from electric neuronal activity) and thus approximately estimate the source based on extracranial electrical measurements [37]. The software computes electrical activity as a current density (μA/mm^2^) without assuming a predefined number of active sources. The LORETA-KEY software divides the neocortical Montreal Neurological Institute (MNI)-152 volume [38], including the hippocampus and anterior cingulate cortex, into 6,239 voxels with dimensions of 5 × 5 × 5 mm. Scalp electrode coordinates on the MNI brain are derived from the international 5% system [39]. Performing 5,000 random permutations, correction for multiple testing (i.e., for the tests performed for all scalp electrodes and/or voxels, and for all time samples and/or discrete frequencies) is carried out and thus no further correction for multiple comparison is needed. The sLORETA algorithm assumes related orientations and strengths of neighboring neuronal sources, and thus the inverse problem is corrected. Anatomical labeling of significant clusters was performed automatically with an embedded toolbox within LORETA-KEY, and these labelings were reconfirmed by reference to the Talairach and Tournoux atlas [40].

### Functional connectivity analysis

Phase synchronization among multiple frequency bands has been suggested to be the most plausible mechanism of large-scale neuronal integration to overcome distributed anatomical and functional organization of brain activity to enable coherent behavior and cognition [41]. Coherence and phase synchronization are interpreted as “connectivity” between distant anatomical locations. Measures of linear dependence (coherence-type) between two multivariate time series may be expressed as the sum of the lagged linear and instantaneous linear dependences. However, measurements of instantaneous dependence are highly contaminated with an instantaneous, non-physiological contribution due to volume conduction and low spatial resolution [42]. To resolve this problem, a refined technique (i.e. Hermitian covariance matrices) that removes this confounding factor considerably has been introduced by Pascual-Marqui [43]. As such, this measure of dependence can be applied to any number of brain areas jointly, i.e. distributed cortical networks, whose activity can be estimated with LORETA-KEY. Accordingly, we calculated lagged linear connectivity (lagged coherence) for the same frequency bands as used for the LORETA-KEY analysis.

For the functional connectivity analysis, 28 ROIs, defined by Brodmann area (BA), were selected as possible nodes, based on previous reports on hyperacusis (Table 2) [17]. Each ROI consists of a single voxel (the one that is closest to the center of mass of the ROI) in LORETA-KEY; thus, the radius around each centroid is 5 mm. Functional connectivity was calculated for all of the eight frequency bands above.

**Table 2:** Twenty-eight regions of interest and their references.

Regions of interest	BA	References
Auditory cortex	41L	[14–17]
	41R	
	42L	
	42R	
	21L	
	21R	
	22L	
	22R	
Insula	13L	[15]
	13R	
Dorsal anterior cingulate cortex	24L	[17]
	24R	
Pregenual anterior cingulate cortex	32L	[17]
	32R	
Subgenual anterior cingulate cortex	25L	[17]
	25R	
Posterior cingulate cortex	31L	[15]
	31R	
Parahippocampus	27L	[15, 17]
	27R	
	29L	
	29R	
orbitofrontal cortex	10L	[15, 17]
	10R	
	11L	
	11R	
Precuneus	7L	[15]
	7R	

BA, Brodmann area; L, left; R, right.

### Region of interest (ROI) analysis

For 2 ROIs that showed statistically significant difference between the hyperacusis group and control group, log-transformed electric current density was averaged across all voxels. Each ROI was defined by a single voxel that was closest to the center of the area where a significant difference was found in the source localization analysis. Each hyperacusis subjects’ log-transformed electric current density in each ROI was then compared to the mean value of 231 control subjects at the same ROI to further evaluate if the results from the source localization analysis were affected by small numbers of hyperacusis and control subjects.

### Statistical analysis

To identify potential differences with regard to resting-state ongoing cortical oscillatory activity, between the five hyperacusis subjects and five normal controls, nonparametric statistical analyses of LORETA-KEY images (statistical non-parametric mapping; SnPM) were performed for each contrast using LORETA-KEY’s built-in voxel-wise randomization tests (5,000 permutations) and employing a *t* statistic for independent groups with a threshold of *P* < 0.01 (corrected for multiple comparison). We have adopted relatively strict threshold for the statistical significance as the number of subjects were relatively small and thus we tried not to include chance-level significant findings from the current study subjects. A correction for multiple comparisons in SnPM using random permutations has been shown to yield similar results with those obtained from a statistical parametric mapping approach using a general linear model with multiple comparisons corrections [44].

For lagged connectivity differences, we compared differences between the hyperacusis group and the normal control group for each contrast using the *t*-statistics for independent groups with a threshold of *P* < 0.05; we also corrected for multiple comparisons using LORETA-KEY’s built-in voxel-wise randomization tests, for all of the voxels included in the 28 ROIs for the connectivity analysis (5,000 permutations).

## Results

### Source-localized group comparison

**Fig 1:**
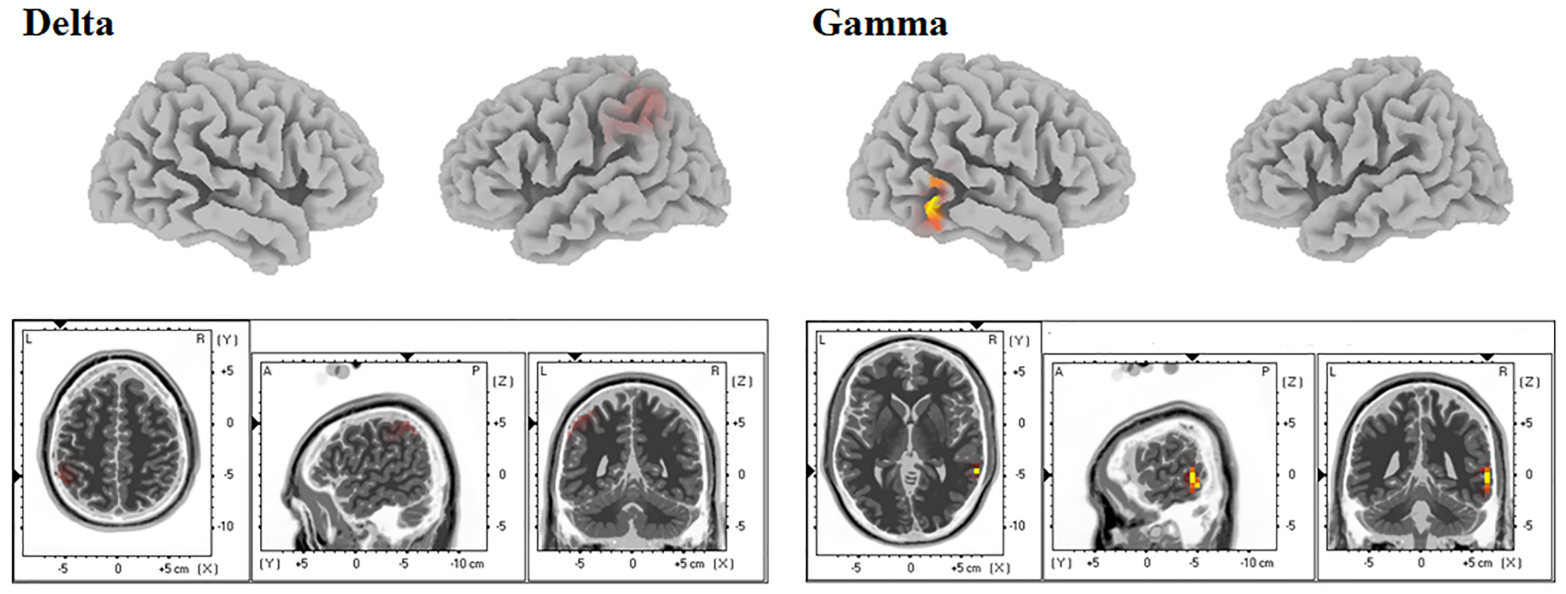
Low-resolution brain electromagnetic tomography (LORETA)-KEY contrast analysis between the hyperacusis group and normal control group. The hyperacusis group demonstrated significantly increased activities in the right auditory-related cortex for the gamma frequency band and left SPL for the delta frequency band as compared with the normal control group (*P* < 0.01).

Compared with the normal control group, the hyperacusis group demonstrated significantly higher electric activities in the left superior parietal lobule (SPL, BA7) for the delta frequency band (*P* < 0.01) and right auditory-related cortex (BA21) for the gamma frequency band (*P* < 0.01; Fig 1). For other 6 frequency bands (theta, alpha 1 and 2, beta 1, 2, and 3), there were no significant differences between the 2 groups for the threshold *P* < 0.01.

### Functional connectivity

**Fig 2:**
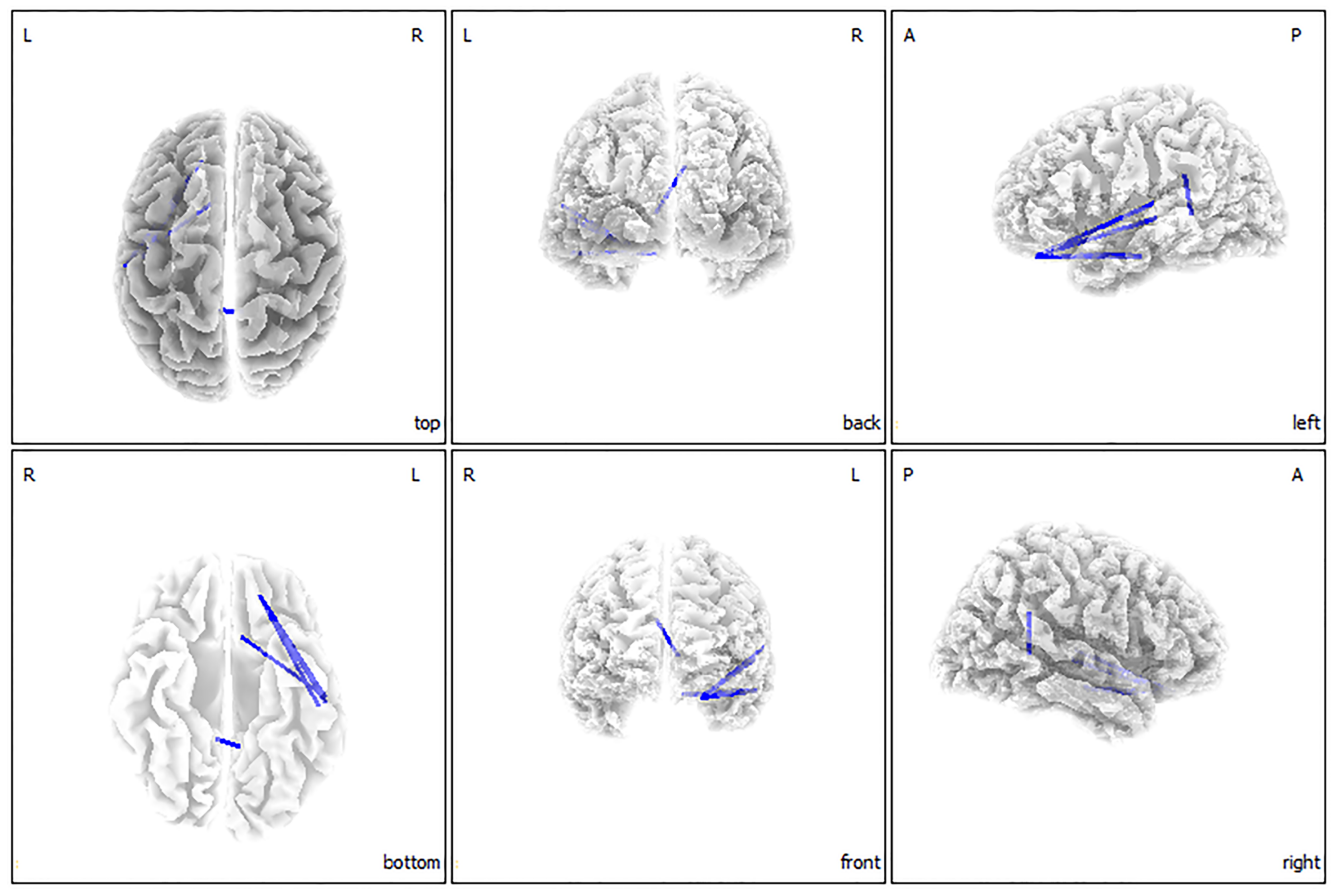
Functional connectivity contrast analysis between the hyperacusis group and normal control group. The hyperacusis group showed significantly decreased functional connectivity as compared with the normal control group between the left A1/A2 and left OFC and between the left A1 and left sgACC for the gamma band.

**Fig 3:**
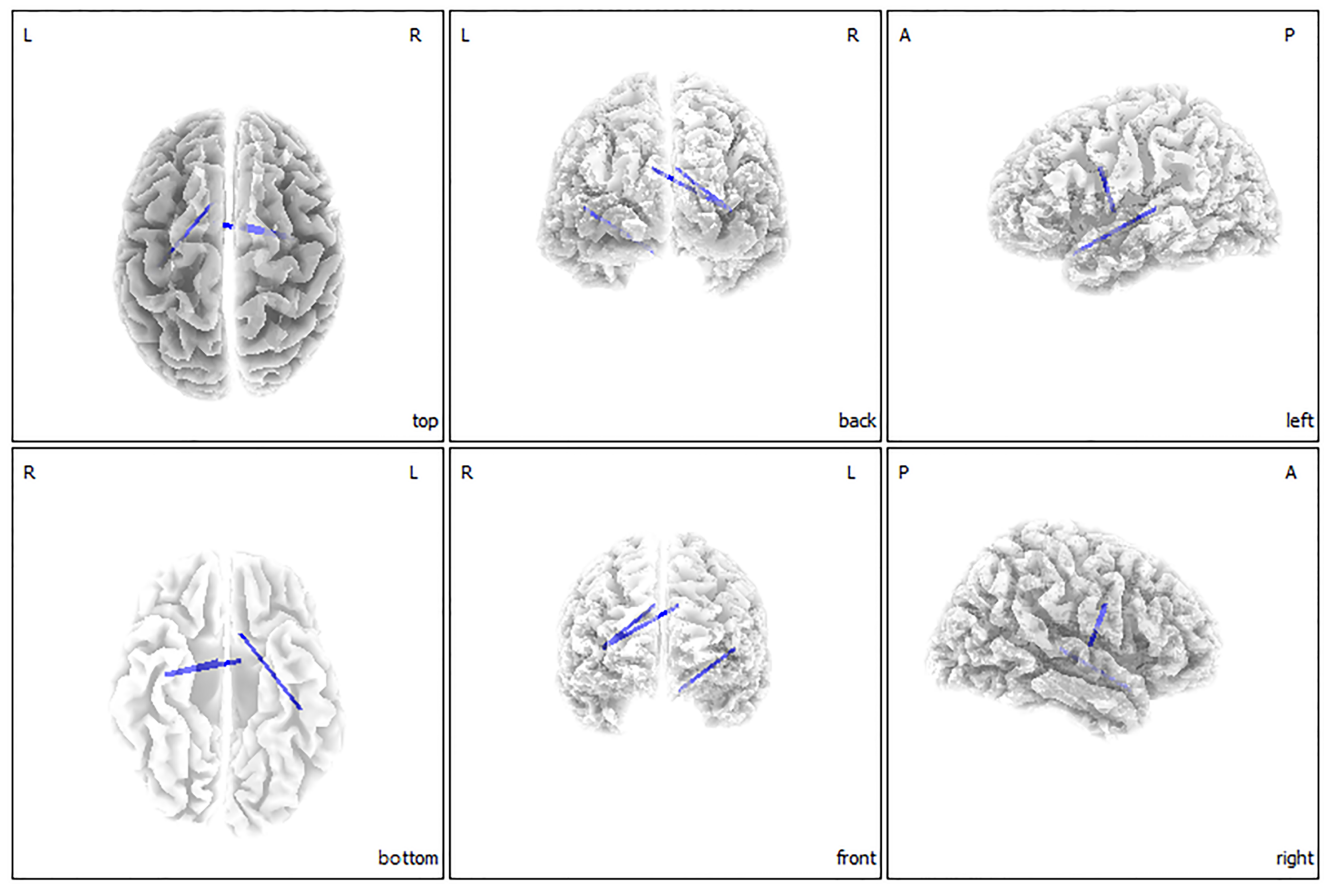
Functional connectivity contrast analysis between the hyperacusis group and normal control group. The hyperacusis group showed significantly decreased functional connectivity as compared with the normal control group between the right insula and bilateral dACC and between the left A2 and left sgACC for the theta band.

The functional connectivity analysis showed significant differences between the hyperacusis patient group and the normal control group for the gamma and theta frequency bands (*P* < 0.05). The hyperacusis group showed significantly decreased functional connectivity versus the normal control group, between the left primary/secondary auditory cortices (A1/A2) and left OFC, and between the left A1 and left subgenual anterior cingulate cortex (sgACC) for the gamma frequency band (Fig 2). Additionally, significantly decreased functional connectivity was found in the hyperacusis group versus the control group between the right insula and bilateral dACC, and between the left A2 and left sgACC for the theta frequency band (*P* < 0.05) (Fig 3). For the other 6 frequency bands, no significant difference was found between the groups.

### ROI analysis

The mean log-transformed current density in the left SPL for the delta frequency band and in the right auditory-related cortex for the gamma frequency band were 1.63 ± 0.46 and 1.08 ± 0.68, respectively, while those of the hyperacusis group were 1.78 ± 0.47 and 1.31 ± 0.40, respectively. The comparison between the 2 groups did not yield any statistical significance due to unequal variance. However, individual comparison showed that 4 of 5 hyperacusis subjects mean log-transformed current density in the left SPL for the delta frequency band and in the right auditory-related cortex for the gamma frequency band were higher than the mean value of the control group.

## Discussion

Previous studies on hyperacusis have been limited in that subjects had comorbidities, such as William’s syndrome and tinnitus. Thus, in the current study, we aimed to identify purely hyperacusis-related neural substrates using rs-qEEG data of patients with idiopathic hyperacusis. Our data demonstrate that the hyperacusis group has significantly increased activities in the left SPL for the delta frequency, and in the right auditory-related cortex for the gamma frequency bands versus the control group. A functional connectivity analysis further showed that the hyperacusis group has significantly decreased connectivity versus the control group, between the left A1/A2 and left OFC, and between the left A1 and the left sgACC for the gamma frequency band (Fig 2). Between the right insula and bilateral dACC, and between the left A2 and left sgACC, decreased connectivity was also seen for the theta frequency band (Fig 3). This suggests that even without sound stimuli, subjects with hyperacusis showed unique cortical oscillatory patterns.

### The auditory cortex may be already hyperactive even without sound stimuli

Several previous studies have reported hyperresponsivity of the AC to repeated auditory stimuli in salicylate- or noise-induced animal models of hyperacusis [12–14]. Moreover, in human studies, elevated sound-evoked activation in the AC was seen in patients who had diminished sound-level tolerances [16]. This hyperactivity to sound stimuli in the AC has been explained in terms of maladaptive brain plasticity and neural network changes [10]. That is, the cortical oscillatory patterns during the resting state, with no sound stimulus, may show changes in cortical activity and functional neural networks in hyperacusis patients [17]. Previous EEG studies on tinnitus [36] or noise trauma [45], conditions in which the AC becomes hyperactive in positron emission tomography functional magnetic resonance imaging studies [34, 46], have indicated that an enhancement in the gamma frequency band in the AC are found. In this regard, the current findings are consistent with our hypothesis, in that we found increased gamma frequency band activity in the auditory-related cortex in hyperacusis subjects versus normal controls (Fig 1). That is, even in the resting state, with no sound-evoked activity, the AC of the subjects with hyperacusis was already hyperactive. The following interpretations of other results may help to explain why this is so.

### A prepared circuit breaker: Higher electrical activity in the SPL

In a recent study on aging, event-related delta responses in the parietal cortex were found to be decreased in accordance with age [47]. Also, another previous study on schizophrenia has revealed increased delta power in schizophrenia patients as compared with normal controls [48]. In this regard, higher electrical activity in the SPL for the delta frequency band in hyperacusis subjects may designate abnormally activated SPL.

When a relevant sensory stimulus is presented, the inferior parietal lobule (IPL) is believed to send a bottom-up ‘circuit breaker’ signal to the SPL [49], which shifts attention to the previously unattended stimulus by top-down attentional modulation. In a previous report on a cochlear implant (CI) user with conversion deafness, the subject showed increased glucose metabolism in the IPL and SPL after recovery from conversion deafness as compared with before recovery [50]. Therefore, higher electrical activity in the SPL under resting-state without any sound stimuli in the hyperacusis group may designate abnormally activated circuit breaker, probably toward forthcoming auditory stimuli. That is, a pre-stimulus enhancement of the circuit-breaking activity mediated by the SPL and resultant over-attention to auditory stimuli may render hyperacusic subjects prone to overreact to ordinary sound stimuli.

### Dysfunctional salience network integrity resulting in abnormal hyperresponsiveness to forthcoming sound stimuli

The hyperacusis group showed significantly decreased functional connectivity between the right insula and bilateral dACC for the theta frequency band (Fig 3). The insula and dACC are known to be component parts of the salience network [18, 19], which is activated only when behaviorally relevant information is processed [18, 20]. In particular, the integrity of the salience network has been suggested to be important for the efficient regulation of activity in the default mode network [19, 51], which is active when a person is not focused on the outside world and the brain is at wakeful rest. That is, the integrity of the salience network is important for filtering forthcoming sensory stimuli and switching from a wakeful resting state to a state of attending to relevant information. In this regard, the decreased functional connectivity between the insula and dACC may indicate a dysfunctional salience filtering system and, thus, abnormal processing of sensory stimuli would be expected.

Indeed, disrupted integrity of the salience network may have resulted in hyperresponsiveness to ordinary sound stimuli in the hyperacusis group, so that *all* sound stimuli become seemingly behaviorally relevant or important. The hyperacusis group showed significantly decreased functional connectivity versus the control group between the left sgACC and left A2 for the gamma frequency band (Fig 2), which may indicate dysfunctional salience filtering for forthcoming auditory stimuli; thus, the subject may lose auditory salience specificity, in that ordinary sound stimuli become perceived as important.

Additionally, the functional connectivity between the left A1/A2 and left OFC for the gamma band was decreased significantly in the hyperacusis group versus the normal controls (Fig 2). Previous studies have indicated that salient environmental events should be perceived preferentially and one means of achieving this is to enhance attention by emotion, leading to the increased detection of salient events [52]. In this regard, decreased functional connectivity between the OFC and auditory cortices may reveal dysfunctional emotional weight attached to auditory stimuli, so that all kinds of sounds are perceived as being salient by the subject. This is also consistent with a previous study on tinnitus subjects with hyperacusis who showed higher electrical activity of the OFC in resting state recordings [17].

Dysfunctional salience network may evoke other related sensory dysfunctions. For instance, a previous meta-analysis on allodynia and hyperalgesia (exaggerated response to innocuous or minimally aversive somatic stimuli) showed activation of the components of the salience network such as the insula and the ACC [53]. As allodynia and hyperalgesia have been frequently compared to hyperacusis based on the analogy of exaggerated response to non-noxious stimuli, future studies on possible hyper-responsiveness to somatic stimuli in hyperacusis subjects may be of importance.

One discrepancy between source-localized group comparison and lagged linear connectivity analysis should be addressed. The current study revealed statistically significant difference in the delta frequency band on source-localized group comparison while decreased functional connectivity was found in the theta frequency band. Although we found differences in different frequency bands, low frequency bands (delta and theta bands) abnormalities are commonly observed in event-related potential studies on cognitive tasks [54] or on emotional processing [55], or in pathologic conditions such as schizophrenia [56]. In this regard, group differences found in different frequency bands in the current study may designate low frequency neural oscillatory abnormalities in subjects with hyperacusis. Future studies in a large series should be conducted to further address this discrepancy.

In short, in a resting state with no auditory stimulus, the salience network of hyperacusis subjects showed dysfunctional integrity, resulting in decreased salience specificity and even abnormal emotional weight being given to forthcoming ordinary auditory stimuli, so that ultimately, the subject suffers from overresponsiveness to sounds.

### Limitations of the current study and proposed future studies

To our knowledge, this is the first reported human functional imaging study addressing possible pre-stimulus neural substrates that may explain the generation of idiopathic isolated hyperacusis. As several culprit cortical areas for hyperacusis have been found, future neuromodulation studies using transcranial magnetic stimulation or transcranial direct current stimulation may be feasible.

Although we found several important cortical oscillatory patterns that may explain the overresponsiveness to sound in subjects with idiopathic hyperacusis, there are several limitations that should be addressed in future studies. First, because the term “hyperacusis” itself represents an abnormal sound-driven psychoacoustic phenomenon, future studies exploring both resting state and sound stimulus-driven cortical activities should be performed to further examine changes in the cortical oscillatory pattern. Second, although idiopathic hyperacusis patients with no other auditory symptom or comorbidity are not frequently encountered, future follow-up studies with larger numbers of subjects should be conducted to replicate the current findings. Our ROI analysis showed that 4 of 5 hyperacusis subjects mean log-transformed current density in the left SPL for the delta frequency band and in the right auditory-related cortex for the gamma frequency band were higher than the mean value of the control group, but still the comparison between the hyperacusis group and 231 control subjects with regard to the mean current density of the ROIs did not reach any statistical significance. Therefore, to further validate that the current results are not affected by a few extraordinary subjects, future follow-up studies are mandatory. Third, an intra-individual study, before and after symptom improvement, should be performed to assess cortical activity changes after treatment in subjects with hyperacusis.

## Conclusions

Taken together, our data showed significantly increased source-localized activities in the right auditory-related cortex and left SPL, as well as decreased functional connectivity between the dACC and insula, and between the auditory cortices and dACC/OFC in hyperacusis subjects versus normal controls in a resting state with no sound stimulus. That is, an abnormally active circuit-breaker and disrupted integrity of the salience network resulted in nonspecific, salient perception of forthcoming sound stimuli; thus, the subject becomes hyperresponsive to ordinary sounds. Developing a neuromodulation treatment strategy targeting these areas may be worthwhile.
